# Laboratory diagnosis of melioidosis

**DOI:** 10.1371/journal.pntd.0013761

**Published:** 2025-12-04

**Authors:** Ian Gassiep, Claire Chewapreecha, Narisara Chantratita, Tessa Oakley, Chiranjay Mukhopadhyay, Piyush Behari Lal, David AuCoin, Fazle Rabbi Chowdhury, Ella M. Meumann, Bart J. Currie, David A. B. Dance, Vanaporn Wuthiekanun, Robert Norton

**Affiliations:** 1 Faculty of Medicine, UQ Centre for Clinical Research, The University of Queensland, Royal Brisbane & Women’s Hospital, Brisbane, Australia; 2 Department of Infectious Diseases, Mater Hospital Brisbane, Brisbane, Australia; 3 Pathology Queensland, Royal Brisbane & Women’s Hospital, Brisbane, Australia; 4 Mahidol-Oxford Tropical Medicine Research Unit, Mahidol University, Bangkok, Thailand; 5 Department of Clinical Tropical Medicine, Faculty of Tropical Medicine, Mahidol University, Bangkok, Thailand; 6 Centre for Tropical Medicine and Global Health, Nuffield Department of Medicine, University of Oxford, Oxford, United Kingdom; 7 Parasites and Microbes Programme, Wellcome Sanger Institute, Hinxton, United Kingdom; 8 Department of Microbiology and Immunology, Faculty of Tropical Medicine, Mahidol University, Bangkok, Thailand; 9 Global and Tropical Health Division, Menzies School of Health Research, Charles Darwin University, Dili, Timor-Leste; 10 Department of Microbiology and Center for Emerging and Tropical Diseases, Kasturba Medical College, Manipal, Manipal Academy of Higher Education, Manipal, India; 11 Department of Microbiology and Immunology, University of Nevada, Reno, School of Medicine, Reno, Nevada, United States of America; 12 Department of Internal Medicine, Bangladesh Medical University, Dhaka, Bangladesh; 13 Global and Tropical Health Division, Menzies School of Health Research, Charles Darwin University, Darwin, Australia; 14 Department of Microbiology, Territory Pathology, Darwin, Australia; 15 Department of Infectious Diseases, Royal Darwin Hospital, Darwin, Australia; 16 Lao-Oxford-Mahosot Hospital-Wellcome Trust Research Unit, Microbiology Laboratory, Mahosot Hospital, Vientiane, Laos; 17 Faculty of Infectious and Tropical Diseases, London School of Hygiene and Tropical Medicine, London, United Kingdom; 18 Faculty of Medicine, The University of Queensland, Brisbane, Australia; National University of Singapore, SINGAPORE

## Abstract

Melioidosis, caused by the Gram-negative bacterium *Burkholderia pseudomallei*, is an infectious disease with high rates of morbidity and mortality, which primarily affects low- and middle-income countries in South and Southeast Asia and Australia. The clinical manifestations of this disease are nonspecific and, therefore, rapid laboratory diagnosis is especially critical as appropriate management requires specific antimicrobials. This article aims to provide an overview of the current diagnostic methodologies, emerging technologies, susceptibility testing, and future perspectives for laboratory diagnosis of melioidosis. By examining conventional culture methods, mass spectrometry, antimicrobial susceptibility testing, antigen detection, molecular diagnostics, and serological assays, this article highlights the current challenges in accurately and cost-effectively diagnosing melioidosis in diverse clinical and resource-limited settings. A detailed analysis of current and future diagnostic methodologies will offer valuable insights for clinicians, researchers, and public health professionals. This review aims to influence clinical and laboratory guidelines for diagnosing melioidosis and future research directions.

## Author summary

Melioidosis is a potentially fatal infectious disease caused by the Gram-negative bacterium *Burkholderia pseudomallei*, found in tropical and subtropical regions worldwide. Infection presents with diverse symptoms, including pneumonia, organ abscesses, and sepsis, which can lead to death within 1–2 days if not promptly diagnosed and treated. Rapid and accurate diagnosis is, therefore, lifesaving. The current diagnostic gold standard is culture, although this method typically requires 2–4 days for results and may suffer from limited sensitivity, particularly in patients who have received prior antibiotic treatment. Several alternative diagnostic approaches exist, including serological tests that detect antibodies against *B. pseudomallei*, and molecular or protein-based assays that identify bacterial components directly from clinical specimens such as sputum, urine, pus, or blood. This review outlines the respective merits and challenges of these diagnostic techniques. As targeted therapy is vital, reliable antibiotic susceptibility testing is also essential, although current techniques may yield inconsistent interpretation. Given that melioidosis primarily affects low- and middle-income countries with limited resources and constrained healthcare infrastructure, there is a pressing need for affordable and practical diagnostic solutions.

## Introduction

Melioidosis, caused by the environmental organism *Burkholderia pseudomallei*, is a life-threatening infectious disease that primarily affects populations in Southeast Asia, South Asia, and Northern Australia [1]. The regions of known endemicity are expanding, through increased recognition with improved diagnostics but also through global spread and unmasking of *B. pseudomallei* in the context of severe weather events. In addition, the prevalence of the most common risk factor for infection, diabetes mellitus, continues to rise worldwide [2]. Known as a “great mimicker,” melioidosis presents with a wide spectrum of symptoms that can resemble other illnesses such as community-acquired pneumonia, tuberculosis, sepsis, pyoderma, and even malignancy [2]. This lack of pathognomonic signs and symptoms, along with variability in presentations hinders clinical diagnosis, making accurate laboratory identification crucial for timely diagnosis and treatment.

The traditional laboratory diagnosis of melioidosis relies heavily on axenic culture from clinical specimens including blood, sputum, pus, and urine. Currently, culture is considered the gold standard for diagnosis, however sensitivity of culture is low, the incubation time is long, and the organisms isolated may be misidentified [3]. Safety and handling of *B. pseudomallei* is an additional consideration due to the risk of laboratory-acquired infection.

Indirect diagnosis with serology has been commonplace in endemic regions for decades. Unfortunately, as with most serological assays, these may not differentiate past exposure from current clinical disease (i.e., melioidosis). Accordingly, methods for direct detection of *B. pseudomallei* antigens or nucleic acids from patient samples have emerged and are promising.

Antimicrobial susceptibility testing of *B. pseudomallei* isolates remains a challenge for the laboratory scientist and microbiologist. While standards for susceptibility testing exist, the availability of broth microdilution, gradient diffusion strips, and even antimicrobial diffusion discs may be limited or unaffordable, especially in the areas where melioidosis is endemic. The vast majority of primary isolates of *B. pseudomallei* have a standard susceptibility pattern which enables confidence in standard treatment regimens for those diagnosed with first-episode melioidosis [4,5]. Therefore, capacity for accurate susceptibility testing for *B. pseudomallei* is a lower priority than capacity for accurate identification of *B. pseudomallei*. Nevertheless, acquired resistance to all standard antimicrobials used for melioidosis has been documented, making susceptibility testing important for any cases with recrudence or relapse after therapy of the first-episode [6].

This review aims to provide the reader with a practical understanding of the laboratory diagnosis of melioidosis.

## Methods

A narrative literature review was conducted to explore current diagnostic approaches for *B. pseudomallei*. Relevant medical literature was identified through focussed searches on PubMed and Google Scholar, complemented by expert-driven inclusion of references considered most applicable to the subject matter. Inclusion criteria were based on clinical relevance, methodological quality, and expert consensus rather than a formal systematic framework. This method enabled a practical and comprehensive overview of diagnostic strategies and challenges encountered in endemic regions. S1 Table details sensitivity and specificity metrics extracted from cited research.

### Laboratory safety

Appropriate handling of *B. pseudomallei* in the laboratory is critical to staff safety. To date, there have been 2 published cases of laboratory-acquired melioidosis, both from the last century [7,8], however, four cases of possible laboratory-acquired infection in an endemic area have also been reported to one of the authors (DABD - Balaji V, personal communication).

There is substantial variation in laboratory physical containment used for *B. pseudomallei* globally. In the USA, *B. pseudomallei* is a Tier 1 select agent and suspected isolates can only be handled in a level 3 biosafety (BSL-3) facility [9]. This immediately limits access to rapid isolate identification in a routine diagnostic laboratory and therefore has the potential to delay diagnosis and lead to patient harm. As the USA has recently been recognised as a melioidosis-endemic region [10], altering these restrictions seems sensible as new cases and foci of melioidosis in the USA continue to emerge [11].

In Australia, the organism is a Risk Group 2 pathogen requiring BSL-2 containment [12], although the use of a class II biosafety cabinet (BSC) is recommended. In Townsville, where the first biosafety precautions for handling of *B. pseudomallei* isolates were published, the organism has been handled on an open bench for decades. A serosurvey of 65 laboratory scientists in 1992 revealed a prevalence of 5% with no historically documented local laboratory-acquired infections [13]. Therefore, the local laboratory practice recommendations included open bench processing of clinical samples except sputum (due to the potential risk of *Mycobacterium tuberculosis* exposure), and open bench handling for standard microbiological processes required for organism isolation and identification. A follow-up serosurvey of 30 laboratory staff who regularly handle *B. pseudomallei* isolates reported 0% seroprevalence [14]. In the intervening 27 years between these serodiagnostic evaluations there were no reported cases of laboratory-acquired infection. *B. pseudomallei* is also routinely handled on the open bench in Darwin, Australia, where ~20–90 melioidosis cases are diagnosed annually; over at least the last 35 years there have been no melioidosis cases among laboratory personnel.

As *B. pseudomallei* can be readily isolated using standard bacterial culture media and growth conditions, and can be challenging to identify, it is likely that this organism will continue to be inadvertently handled on the open bench. Furthermore, handling of all cultures in a biosafety cabinet in BSL-3 containment is impractical, and is likely to lead to delays in organism identification and timely antimicrobial treatment. Given the rarity of laboratory-acquired infections, we recommend local risk assessment based on melioidosis incidence, staff experience and familiarity with *B. pseudomallei*, available resources and impacts on laboratory workflow, and available risk mitigation strategies. Where staff are exposed to *B. pseudomallei*, assessment and management of this should take into account staff comorbidities and immune status, and the nature of the exposure including whether it involved potential aerosolization or percutaneous inoculation of *B. pseudomallei* [9]. Post-exposure prophylaxis with trimethoprim-sulfamethoxazole may be offered, however, importantly this can be associated with serious side effects [15,16].

### Culture

**Fig 1:**
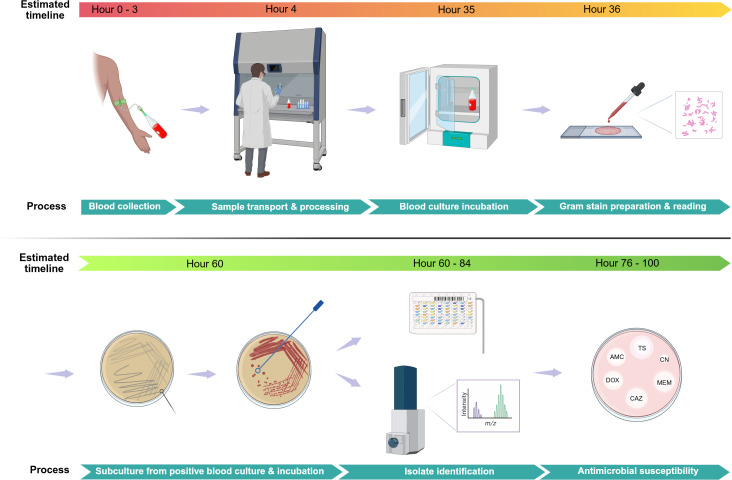
Routine diagnostic workflow for *B. pseudomallei* bloodstream infection: From sample collection to susceptibility testing. Created in BioRender. Gassiep, I. (2025) https://BioRender.com/5209mfb. Blood cultures are collected aseptically at the patient’s bedside. The inoculated bottles are transported promptly to the microbiology laboratory under appropriate conditions and loaded into an automated blood culture incubator system for continuous monitoring and incubation (where automated systems are not available visual inspection and blind subculture are required, delaying identification by days). Upon detection of a positive signal, an aliquot of the blood culture broth is removed for Gram staining, which provides a preliminary report of the organism’s morphology and Gram reaction. Simultaneously, subcultures are made onto suitable solid media for isolation of pure colonies. While not routinely performed, organism identification can be achieved directly from positive blood culture broth using antigen detection, proteomic, or molecular techniques. Following incubation, discrete colonies obtained from subcultures are subjected to organism identification using a biochemical identification systems, latex agglutination, or mass spectrometry. Antimicrobial susceptibility testing is then performed on the identified isolate using disk diffusion. Notably, on receipt in the laboratory, nonblood specimen are directly inoculated onto solid media and therefore, organism identification may occur 24–48 hours sooner.

Culture and identification of *B. pseudomallei* remains the gold standard for the diagnosis of melioidosis, although modelling from Thailand suggested its sensitivity for *B. pseudomallei* identification may be as low as 60% [3]. In the context of adequate laboratory culture capacity and repeat sampling and culture, sensitivity of culture should be well over 90%. However, prior antibiotic exposure may reduce culture sensitivity, and culture-negative cases are typically restricted to infection in sites not amenable to tissue sampling, such as brainstem and spinal cord [17]. *B. pseudomallei* is not part of the normal human microbiota, so its presence in any sample confirms melioidosis. This relies on clinicians considering the diagnosis and sending the right specimens from the right patients. Blood cultures are the most important, as bacteraemia is common, occurring in 56–70% of melioidosis cases, Fig 1 [18]. Other samples to culture include pus from abscesses, sputum in patients with pneumonia, and urine from patients with suspected urinary tract involvement (e.g., prostatic abscess). Culture of the centrifuged urine pellet may increase sensitivity for *B. pseudomallei* identification, and in Darwin, Australia, where prostatic abscess is a common melioidosis presentation, this is conducted on all urine specimens from men with pyuria during the wet season. In some centres, throat swabs, and rectal swabs are recommended for suspected cases and can be useful where the patient is unable to produce sputum, although selective media are needed for optimal sensitivity with such samples [19,20]. These samples may be the only ones to test positive in some patients, especially those with liver or splenic abscesses that cannot be aspirated [21]. Clinical samples should be transported to the laboratory at room temperature and processed as soon as possible [22].

*B. pseudomallei* grows well on most routine laboratory media, including commercial blood culture broth, Brain Heart Infusion broth, Trypticase Soy Broth, and blood, chocolate, and MacConkey agars. However, it grows slowly compared with many other pathogens and colonies may only be pinpoint-sized after 24–48 hours of incubation at 35 °C. *B. pseudomallei* can be overlooked by the unwary, either due to overgrowth by other flora or because its colonial morphology is mistaken for a contaminant. Selective media that have been developed specifically for the isolation of *B. pseudomallei* from nonsterile sites include Ashdown’s agar and broth, Francis medium, and *B. pseudomallei* selective agar [23–25]. These media contain ingredients that support the growth of *B. pseudomallei* and induce characteristic colonial features while suppressing other organisms. Incubation for up to 4 days is sometimes necessary, particularly for samples with a low bacterial load, resulting in a total diagnostic time of 6 days or more (2 days for initial culture plus an additional 2 or more days on selective media) [2,26,27]. Where these media are not available, selective media for *B. cepacia* may be useful [28]. Ashdown’s agar and selective broth are simple, easy to prepare, and cost-effective [29], although because it contains gentamicin, Ashdown’s agar is not appropriate for use in areas where gentamicin-susceptible *B. pseudomallei* has been found such as parts of Malaysia [30].

Although bacterial culture is regarded as the gold standard diagnostic method, colony analysis must be performed with caution, as several factors can contribute to false-negative results. For example, the sensitivity and specificity of diagnostic tests performed on positive blood culture broth are inherently dependent on the sensitivity of the initial blood culture. Furthermore, *B. pseudomallei* colonies may sometimes resemble contaminants and thus be overlooked, underscoring the importance of meticulous examination by experienced microbiologists to enhance detection accuracy.

### Organism identification

*Burkholderia pseudomallei* are Gram-negative, rod-shaped bacteria that may exhibit a characteristic ‘safety-pin appearance’. Growth on MacConkey agar is nonpigmented (nonlactose fermenting) or faintly pink colonies. After 48-hours’ incubation on horse blood or chocolate agar colonies demonstrate a white to creamy coloration with metallic-sheen appearance. Presumptive identification of *B. pseudomallei* may be achieved using a combination of biochemical tests, Table 1 [31–33]. However, a straightforward approach combining Gram staining, a bench-top oxidase-positive test, and disc diffusion showing susceptibility to amoxicillin-clavulanate alongside resistance to gentamicin and colistin is highly specific for presumptively identifying *B. pseudomallei* from clinical samples [34].

**Table 1:** Differentiation of *B. pseudomallei* from its closely related clinically relevant species (other *Burkholderia* spp., *Ralstonia* spp., *Stenotrophomonas maltophilia,* and *Pseudomonas* spp.) by biochemical characteristics.

	Oxidase production	Nitrate reduction	OF test (Glucose)	OF test (Sucrose)	Mannitol fermentation	Motility	Lysine decarboxylase test	Arginine dihydrolase test	Colistin sensitivity
*Burkholderia pseudomallei*	**+**	**+**	**A**	**_**	**_**	Motile	**_**	**+**	Resistant
*Burkholderia cepacia*	**+**	**_**	**A**	**V**	**V**	Motile	**+**	**_**	Resistant
*Burkholderia gladioli*	**–**	**V**	**A**	**_**	**_**	Motile	**_**	**V**	Resistant
*Burkholderia thailandensis*	**+**	**+**	**A**	**A**	**_**	Motile	**_**	**_**	Resistant
*Burkholderia mallei*	**V**	**+**	**A**	**V**	**_**	Non motile	**_**	**+**	Resistant
*Ralstonia picketti*	**+**	**+**	**A**	**_**	**_**	Motile	**_**	**+**	Resistant
*Ralstonia insidiosa*	**+**	**+**	**A**	**_**	**+**	Motile	**_**	**_**	Resistant
*Ralstonia mannitolytica*	**+**	**–**	**A**	**_**	**+**	Motile	**_**	**_**	Resistant
*Stenotrophomonas maltophilia*	**_**	**V**	**V**	**V**	**_**	Motile	**+**	**_**	Susceptible
*Pseudomonas aeruginosa*	**+**	**+**	**A**	**_**	**V**	Motile	**_**	**+**	Susceptible
*Pseudomonas stutzeri*	**+**	**+**	**A**	**_**	**V**	Motile	**_**	**_**	Susceptible

OF, Oxidative fermentative; + , Positive; −, Negative; V, Variable; A, Acid reaction; S, Susceptible; R, Resistant.

Note: Negative for OF represents nonsaccharolytic reaction.

Biochemical tests alone are not enough to confirm the identification of the bacterium at the species level. A simple biochemical identification method which incorporates a number of the aforementioned reactions is the Analytical Profile Index (API) 20NE strip (bioMérieux, Marcy-l’Etoile, France). This group of manual biochemical tests is able to provide a phenotypic profile for organism identification in most cases, although interpretation of the carbon source assimilation tests may be difficult and in some hands it has been reported to be unreliable [35]. Alternatively, an automated biochemical analysis instrument such as the Vitek 2 (bioMérieux, Marcy-l’Etoile, France) can also be used for organism identification. Unfortunately, this may also be prone to misidentification, and cannot be reliably used to identify *B. pseudomallei* [36].

Polyclonal and monoclonal antibodies have been used for the identification of *B. pseudomallei* isolates via latex agglutination or immunofluorescence microscopy [37–39]. The preferental development of monoclonal over polyclonal antibodies over time is due to less batch-to-batch variability and improved specificity [39].

Although not currently utilised as a standard identification method in most laboratories, monoclonal antibodies have demonstrated 94–100% sensitivity and 84–100% specificity when applied to a cultured isolate for *B. pseudomallei* identification [37,40–42].

The historical limitations for implementation of a monoclonal assay related to technical challenges and cost of producing high-quality antibodies. Current constraints, predominantly in resource-limited settings, include the need for specialised equipment such as fluorescent microscopes and laboratory expertise. These challenges have largely been overcome with the development of an immunochromatographic lateral flow rapid diagnostic test which can be performed on clinical samples, bacterial colonies, or broth cultures [43].

Identification of *B. pseudomallei* specific DNA from a clinical isolate can confirm the diagnosis of melioidosis. Multiple methods have been developed including polymerase chain reaction (PCR) and loop-mediated isothermal amplification (LAMP) [44]. Despite the highly recombinogenic *B. pseudomallei* genome, a number of gene targets including the type III secretion system (T3SS), have demonstrated 100% sensitivity and specifity for *B. pseudomallei* identification [45–47]. The routine use of PCR for confirmation of *B. pseudomallei* from culture is limited by the requirement for specialised equipment, reagents, and expertise. LAMP offers a simpler, cheaper, and faster alternative to PCR [48]. The DNA amplification is isothermal (performed at room temperature), requires less than 60 min, and detection is performed via visual assessment of turbidity. Although specialised equipment is not necessarily required, the reagents required for a LAMP assay traditionally required storage at −20 °C. Additionally, LAMP assay have previously demonstrated a comparative lack of specificity [48]. These limitatons have been addressed by freeze-drying reagents and improved primer design [49].

In many places mass spectrometry (MS) has largely replaced the traditional biochemical methods of bacterial identification and become a vital diagnostic instrument in the clinical laboratory. MS is a reliable method of *B. pseudomallei* identification, provided spectra for this organism are included in the database in use. There are multiple reports of misidentification by MS of *B. pseudomallei*, mainly as *Burkhoderia thailandensis*, where the database has not included *B. pseudomallei* [50,51]. Likewise, inclusion of representative spectra from geographically diverse *B. pseudomallei* strains is important [52].

Two matrix-assisted laser desorption-ionisation time of flight (MALDI-TOF) MS instruments are currently commercially available. The Bruker Biotyper (Bruker Daltonik GmbH, Bremen, Germany), using the Security-Relevant library, has been able to accurately identify *B. pseudomallei* for over a decade [53]. Unfortunately, the *B. pseudomallei* proteomic spectrum used for organism identification is not included in the standard Bruker database, thus requiring the additional library at an additional cost. The Vitek MS (bioMérieux, Marcy-l’Etoile, France) is widely used in diagnostic laboratories across many melioidosis-endemic regions, and the standard database (version 3.3) released in 2023 now includes *B. pseudomallei* [36].

**Fig 2:**
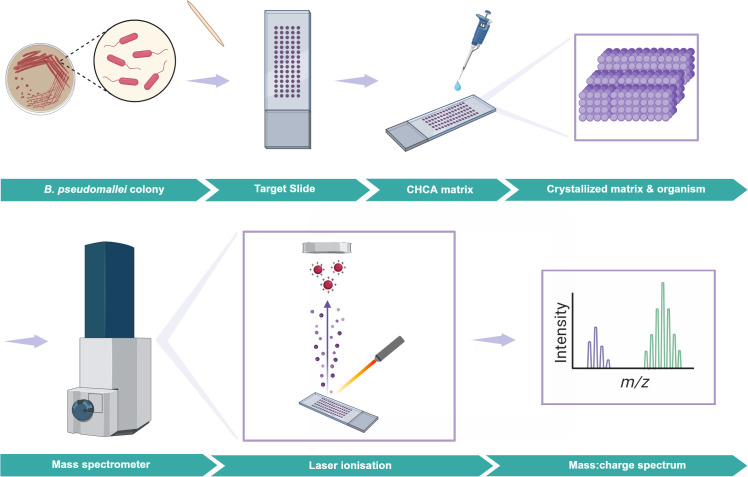
Mass spectrometry. Created in BioRender. Gassiep, I. (2025) https://BioRender.com/xbq39lt. A *B. pseudomallei* colony is picked from an agar plate with a toothpick and placed on a target slide. α-Cyano-4-hydroxycinnamic acid is added and once dry creates a crystallized matrix which both inactivates *B. pseudomallei* and allows for even ionisation. The target slide is placed into the instrument where laser ionisation results in an organism-specific mass-to-charge ratio and relative abundance of ions.

There are a multitude of advantages to using mass spectrometry for *B. pseudomallei* identification. Creation of a target spot on the MS slide is unlikely to result in aerosol generation, and the standard matrix, a-Cyano-4-hydroxycinnamic acid, inactivates the organism once crystallised, Fig 2 [54]. Therefore, the risk to laboratory staff relating to target slide preparation and handling is negligible. Once on the instrument the time to organism identification occurs within minutes. In terms of accuracy, an Australian analysis of the Vitek MS reported 100% organism identification in a validation cohort [36]. Time to organism identification can also be improved by subculturing a positive blood culture onto chocolate agar with an abbreviated 6-hour incubation and subsequent MS analysis [54].

Culture-based antigen detection methods, such as bacterial growth on solid media followed by testing of developed colonies, require facilities equipped with biosafety cabinets, incubators, automated blood culture systems, and MALDI-TOF mass spectrometry where available. For rapid diagnosis, LA, LFA, and IFA enable antigen detection directly from bacterial colonies or broth within 15 min, reducing the time required for presumptive *B. pseudomallei* identification following sample collection from 3–5 days to 1–2 days [55–57].

### Antimicrobial susceptibility testing

The recommended treatment for melioidosis is intravenous ceftazidime or a carbapenem (imipenem or meropenem), followed by oral therapy with trimethoprim-sulfamethoxazole. Amoxicillin-clavulanate and doxycycline are second-line oral therapy options.

Currently, the Clinical and Laboratory Standards Institute (CLSI) recommends a broth microdilution (BMD) method for *B. pseudomallei* susceptibility testing [58]. Additionally, it is important to note that CLSI does not include a clinical breakpoint for meropenem susceptibility testing, but only imipenem.

As of 2020, the European Committee on Antimicrobial Susceptibility Testing (EUCAST) released clinical breakpoints for both a BMD and disc diffusion (DD) method [59] based on the results of a multi-centre study of clinical *B. pseudomallei* isolates [60]. As the majority of *B. pseudomallei* isolates are cultured in low- and middle-income countries, the use of disc diffusion is both practical and more affordable. A notable limitation of disc diffusion is the interpretation of trimethoprim-sulfamethoxazole (TMP-SMX), which in the past has resulted in over-reporting of resistance [61]. The previous recommendation to read the minimum inhibitory concentration at the 80% inhibition point, and the current EUCAST recommendation to read the outer zone edge are both subject to variable inter-operator interpretation. Isolates testing resistant via disc diffusion to TMP-SMX should therefore be re-evaluated with an alternative method. Practically, this is likely to be a gradient diffusion antimicrobial strip such as an ETEST (bioMérieux, Marcy-l’Etoile, France). The reading of TMP-SMX gradient diffusion results still remains challenging due to the double-zone of inhibition [2]. From a laboratory standards perspective, the clinical microbiologist should be aware that *B. pseudomallei* is not currently included within the ETEST Instructions for Use (IFU). Therefore, these results may be considered nonstandardised and potentially reported without an interpretation. In a multi-centre study comparing ETEST with BMD, categorical agreement was ≥97% for ceftazidime, imipenem, doxycycline, and amoxicillin-clavulanate, but was 80% for trimethoprim-sulfamethoxazole [62].

The reporting of susceptibility results has become an additional challenge since EUCAST created the “Susceptible, increased exposure” category, listed as “I”, which was an unfortunate choice as it continues to create confusion amongst clinicians, many of whom have experience in interpreting the CLSI “I” or “Intermediate” category as potentially resistant and “SDD” or “Susceptible Dose-Dependent” category as those requiring “increased exposure”. Aside from including an interpretive comment on laboratory reports (such as ‘Susceptible but requires high doses’), which may not be read, it is important to continue to educate clinicians that a EUCAST “I” equals “S” with the appropriate (increased) antimicrobial dosing [63]. For *B. pseudomallei* specifically, meropenem and imipenem are the only agents for which standard dosing is considered appropriate, and therefore are reported as either “S” or “R” (although resistance is rare). All other agents, including ceftazidime, TMP-SMX, doxycycline (using a tetracycline screening disc), amoxicillin-clavulanic acid, and chloramphenicol are reported as either “I” or “R”, reflecting the higher than standard doses of these antimicrobials needed to treat melioidosis [63].

### Detection of *Burkholderia pseudomallei* antigens directly from clinical or cultured samples

**Fig 3:**
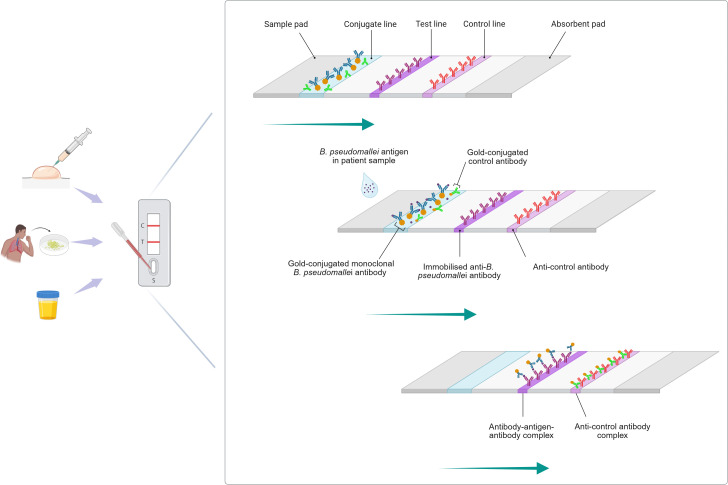
Lateral flow assay. Created in BioRender. Gassiep, I. (2025) https://BioRender.com/o3h78f4. Clinical specimens including pus, sputum, and urine are potential samples for direct analysis. A liquid sample containing *B. pseudomallei* antigens is placed onto the sample pad. The sample is wicked across the nitrocellulose test pad (left to right). The antigen in the sample is bound by mobile antigen-specific gold-conjugated-antibodies which travel across the membrane and are subsequently bound to the immobilised *B. pseudomallei*-specific antibodies on the test strip. The aggregation of this gold-conjugated antibody-antigen-antibody complex is seem as a visible line (colour dependant on conjugate).

Several antigen detection assays have been developed for the rapid identification of *B. pseudomallei* directly in clinical and environmental samples, Fig 3. These include immunofluorescent assay (IFA) [39,55,56], enzyme-linked immunosorbent assay (ELISA) [41,57], electrochemical immunoassays (iSTAT and ECIA) [64], and lateral flow assay/immunoassay (LFA/I) [65–68].

IFA, LFA, ELISA, iSTAT, and ECIA utilise monoclonal or polyclonal antibodies to recognise specific *B. pseudomallei* antigens. The performance of these assays varies depending on several factors, including the target antigen, specimen type, bacterial load, timing of sample collection, and detection methodology.

Antigens targeted by these assays include polysaccharides, proteins, and crude bacterial antigens [38,39,55–57,64–67]. Among them, the most commonly used are capsular polysaccharide (CPS) and lipopolysaccharide (LPS), as they are conserved among *B. pseudomallei* isolates and are divergent from those found in other bacteria [65–67,69–71]. However, more findings highlight certain limitations in using these antigens for antigen detection. For instance, cross-reactivity with closely related *Burkholderia* species can compromise specificity. *B. pseudomallei*, *B. thailandensis*, and *B. mallei* share the same LPS antigen, while some strains of *B. thailandensis*, *B. mallei*, and *B. cepacia* express CPS similar to *B. pseudomallei* [72–74]. Further challenges arise from regional variations in LPS antigenic types (e.g., type A, B, B2, and rough variants), which have been observed across Australia, Southeast Asia, and South Asia, potentially affecting assay performance [75–77]. Additionally, antigenic switching of LPS in response to environmental conditions has been documented [78], necessitating careful interpretation of diagnostic results. Multiple-antigen detection approaches may improve sensitivity and specificity for *B. pseudomallei* identification, enhancing diagnostic accuracy.

The detection limits of various assays differ depending on the type of clinical specimen. IFA and ELISA typically require approximately 10³ colony-forming units (CFU)/mL for detection [39,55–57], whereas latex agglutination (LA) has a higher detection limit of 10⁶ CFU/mL [38]. Sensitivity of antigen detection can be significantly improved to 99–100% when enrichment with a culture step is performed, particularly for samples with low bacterial loads such as blood, throat swabs, and environmental specimens [36,39,55–57,65]. In contrast, samples with higher bacterial loads including pus, sputum, and urine, usually have high sensitivity without a culture step [39,67].

Detection of *B. pseudomallei* antigens directly from patient samples has been challenging. One of the main issues is the low concentration of bacteria that is present in samples, especially blood (~1 CFU/ml in most patients except those who are extremely sick) [79–81]. This issue certainly affected the performance of a lateral flow assay which demonstrated low and variable sensitivity for *B. pseudomallei* identification when testing serum and whole blood samples (14–40%) collected from blood culture-positive patients [43,82]. The LOD of the latest version of the LFI appears to be roughly 1 x 10^5^ CFU/ml and 200–300 pg/ml of shed/secreted CPS [43,66]. Although other specimen types such as sputum and pus can contain higher concentations of bacteria, these are not always available [79,80]. Given the low concentration of *B. pseudomallei* in blood there have been efforts to identify antigens that are shed/secreted from the bacteria, potentially from internal abscesses. The most promising *B. pseudomallei* antigen appears to be the manno-heptose CPS [83]. This antigen appears to be shed into blood at relatively low concentrations but accumulates in urine [83,84]; an analysis of paired serum and urine samples indicated that CPS concentrations are consistently higher in urine than serum [67]. In the same study the lateral flow assay detected CPS in 40.5% of urine samples, compared to 6.5% of serum samples from melioidosis patients analysed, suggesting that urine is a preferable specimen type. Efforts are still underway to improve the analytical sensitivity of the LFI which is roughly 1 x 10^5^ CFU/ml and 200–300 pg/ml of shed/secreted CPS [43,66]. Taken together the lateral flow assay for CPS detection i) performs extremely well on colonies and broth from blood culture bottles that have signalled positive [43], ii) may show promise for sample types that accumulate higher concentrations of *B. pseudomallei* (sputum, pus and serum in patients with severe bacteremic sepsis), iii) demonstrates higher sensitivity when testing urine vs. serum, and iv) may perform substantially better if CPS is pre-concentrated in higher volumes of urine.

Currently, only the lateral flow assay continues to be developed. Of the two electrochemical immunoassays for detecting CPS, the iSTAT showed promise when tested on patient clinical samples [68] but costs were prohibitive and while the ECIA study suggested a low LOD [64], no validation has occurred with testing of clinical samples. The Active Melioidosis Detect (AMD) lateral flow assay remains available for research use only and efforts are ongoing to seek FDA approval [85].

### DNA-based detection methods for *Burkholderia pseudomallei*

**Fig 4:**
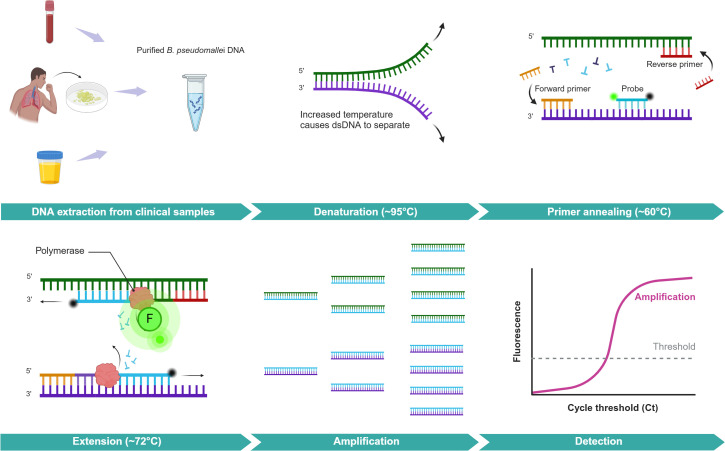
Molecular detection by real-time Polymerase Chain Reaction. Created in BioRender. Gassiep, I. (2025) https://BioRender.com/r9l042c. Direct molecular detection of *B. pseudomallei* from clinical samples involves several steps. After collecting a sample of blood, sputum, or urine, bacterial DNA is extracted using a commercial kit which isolates the DNA and removes inhibiting substances. Next, a real-time PCR assay targeting a specific gene, such as the Type 3 Secretion System 1 (TTS1), is performed. The assay contains nucleotides, DNA polymerase, primers, and probes, and occurs under optimised thermal cycling conditions including denaturation, annealing, and extension. A fluorescent signal, which is inhibited by proximity to a quencher, is released when the probe is hydrolysed. The amount of fluorescence is measured in real-time, and a result is generated once a threshold is overcome.

DNA-based detection methods for *B. pseudomallei* and closely related species have evolved significantly, with polymerase chain reaction (PCR) being the first widely introduced technique, Fig 4 [86–93]. However, in many melioidosis-endemic regions—often resource-limited settings—PCR may not be readily available due to its equipment and infrastructure requirements. To address this limitation, alternative equipment-light isothermal amplification methods, such as loop-mediated isothermal amplification (LAMP) [48,49,94] and recombinase polymerase amplification (RPA) [95,96] have been developed for regional laboratories.

All these amplification techniques rely on the design of primers that target DNA sequences conserved in *B. pseudomallei* but divergent from closely related species [97]. However, primer design poses significant challenges due to the relatively large genome of *B. pseudomallei* (7.2–7.5 Mb) and its high GC content (68–69%), which is considerably higher than that of many other bacteria [98]. While the high GC content contributes to genome stability and adaptability to harsh environments, it also reduces DNA denaturation during amplification due to there being 3 hydrogen bonds between GC interactions (rather than 2 for AT interactions). Furthermore, the genetic diversity and open pangenome of *B. pseudomalle*i can make the identification of conserved target regions challenging [99–102].

Currently, the type three secretion system (TTS1) open reading frame 2 (*orf2*) is the most widely used species-specific gene target [90].

The recent development of molecular diagnostics for melioidosis has focussed on identifying gene targets with high sensitivity and specificity across diverse clinical specimens. Traditionally, the type III secretion system (*TTS1-orf2*) gene cluster has been widely used target for *B. psedomallei* detection by real-time PCR [90]. Recent studies have expanded the repertoire of molecular targets evaluated for diagnostic purposes. Noparatvarakorn et al. optimised real-time PCR assays targeting six genes including *TTS1-orf2, BPSS0745, BPSS0087, BPSS1187, BPSS1498 (hcp1)*, and *BPSS1492 (bimA)* [103,104]. The limit of detection (LOD) was ≤10 genome equivalents (GE)/reaction for *TTS1-orf2, BPSS0745, BPSS1187*, and *BPSS1498*, while BPSS0087 and BPSS1492 had LODs of 100 GE/reaction, suggesting lower sensitivity for the latter two targets.

Clinical evaluation demonstrated that *BPSS1187* outperformed *TTS1-orf2*, particularly in plasma samples. Among bacteremic melioidosis patients, *BPSS1187* exhibited a sensitivity of 76% with 100% specificity for *B. pseudomallei* identification, while in a larger prospective cohort with 705 samples from 421 suspected cases, *BPSS1187* achieved 90% sensitivity compared to 79% for *TTS1-orf2* (*P* = 0.004), with specificities of 96% and 99%, respectively. In plasma alone, *BPSS1187* reached a sensitivity of 85%, significantly higher than *TTS1-orf2* (68%; *P* = 0.007) [103]. These findings suggest that *BPSS1187* is a promising molecular target for early and sensitive detection of *B. pseudomallei* in clinical specimens.

The increasingly extensive and geographically diverse set of publicly available genomic data from *B. pseudomallei* populations provides an opportunity to enhance primer design through *in silico* analyses, improving primer coverage and ensuring high theoretical sensitivity [99–102]. Analytical sensitivity determined with serial dlutions of target DNA has demonstrated a limit of detection as low as single-digit copy numbers [105], although measurement uncertainty may be increased by potential pipette inaccuracy and sample handling inconsistencies, and the actual sensitivity for *B. pseudomallei* identification observed in clinical and environmental samples likely varies due to the presence of PCR inhibitors, such as those found in urine, blood, or laboratory reagents. LAMP and RPA have demonstrated greater tolerance to inhibitors compared to traditional PCR, enhancing their suitability for field applications [106,107].

**Fig 5:**
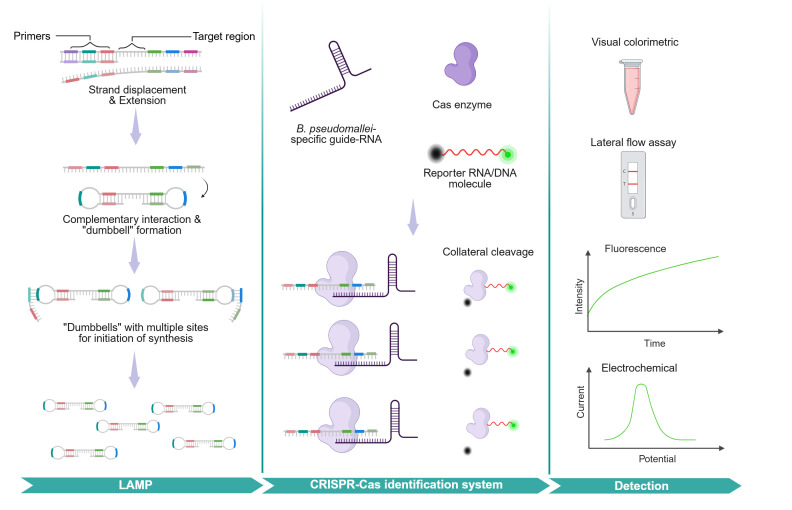
Molecular detection by loop-mediated isothermal amplification and CRISPR-CasCreated in BioRender. Gassiep, I. (2025) https://BioRender.com/r9l042c. Simplified schematic of Loop-mediated isothermal amplification (LAMP) paired with CRISPR-Cas system. LAMP achieves high specificity by using 4–6 primers that recognise multiple distinct regions of the *B. pseudomallei* genome. A strand-displacing DNA polymerase initiates synthesis without thermal denaturation, displacing the complementary strand as it extends. The displaced DNA forms stem–loop structures due to the complementary sequences introduced by the primers, enabling rapid, continuous amplification at a constant temperature. In CRISPR-based diagnostics, a guide RNA (gRNA) is designed to match a specific DNA or RNA target sequence, which in this context, corresponds to the LAMP amplification product. The gRNA directs a Cas nuclease—such as Cas12a for DNA targets or Cas13a for RNA—toward the complementary sequence. Upon binding, the nuclease is activated and exhibits collateral activity, indiscriminately cleaving nearby single-stranded DNA (Cas12a) or RNA (Cas13a). This property is harnessed by adding synthetic reporter molecules that release a detectable signal when cleaved. Detection can be performed using methods such as fluorescence measurement or lateral flow assays.

While isothermal amplification techniques offer promising sensitivity, they are susceptible to nonspecific amplification, which can lead to false-positive results. To improve specificity, recent methods [108–111] have incorporated CRISPR-based detection systems, which leverage crRNA-programmed, DNA-targeting Cas proteins to confirm the presence of the correct amplicon in a sequence-specific manner, Fig 5. A novel biosensor approach incorporating a combination of bacteriophage and CRISPR-based detection may further improve sensitivity and specificity for *B. pseudomallei* identification [112]. These systems offer diverse readout options, including quantitative fluorescence, electrochemical detection, and lateral flow strips, making them suitable for field deployment [108].

The combination of isothermal amplification methods with CRISPR-based confirmation represents a significant advancement in the molecular detection of *B. pseudomallei*, offering both sensitivity and specificity required for accurate diagnosis in diverse settings.

### Antibody detection

**Fig 6:**
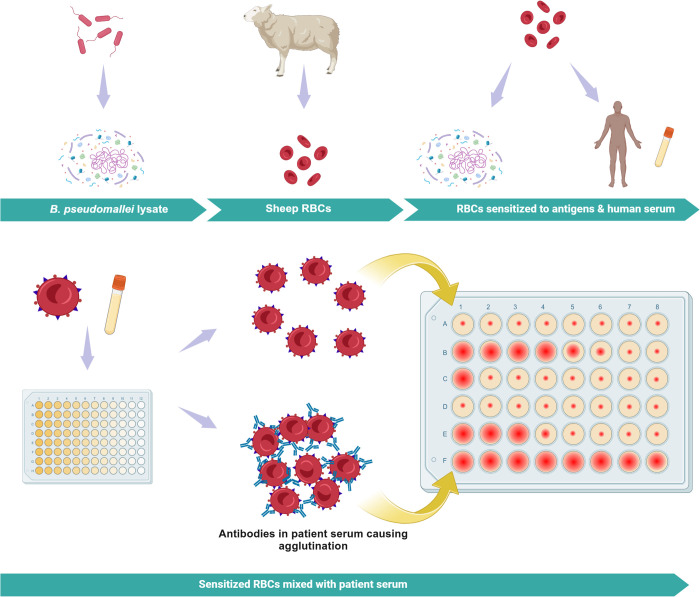
Indirect hemagglutination assay. Created in BioRender. Gassiep, I. (2025) https://BioRender.com/xbq39lt. *B. pseudomallei* isolates are combined to form a crude, nonstandardised, antigen rich lysate. This lysate is used to sensitise sheep red blood cells (RBCs) with *B. pseudomallei* antigens. Patient serum is incubated with nonsensitised sheep RBCs to remove nonspecific agglutinins. The incubated patient sera are diluted by 2-fold serial dilutions in a 96-microtiter plate and antigen-sensitised red cells are then added to the wells. *B. pseudomallei* antibodies present in a patient’s serum will create an antigen-antibody complex, resulting in haemagglutination and the appearance of a hazy or lattice-like structure.

The serological diagnosis of melioidosis is challenging because of the background seropositivity in endemic populations, and poorly characterised antigens used in some tests [21]. Ashdown and Guard in 1984 showed that an average of 5.7% of random individuals from North Queensland had a titre of 1:40 or greater for the melioidosis indirect haemagglutination assay (IHA), Fig 6 [113]. The specificity of the IHA ranges from 64% to 100%, depending upon the cut-off titre, the timing of the sample during the course of infection, and endemicity [114–116]. Some patients with culture-confirmed melioidosis have been shown not to seroconvert in acute illness with a reported sensitivity from Australia of 56%, suggesting a limited diagnostic utility in this setting [117]. However, an IHA titre of greater than 1:320 was very likely to be due to infection with a specificity of 97% [118]. Further, a cut-off of ≥ 1:640 in a low seroprevalence Australian population was used to support the diagnosis of suspected melioidosis in 11% of culture-negative patients [119]. Importantly, this study also provided insights into patients without confirmed melioidosis and a high IHA titre. These patients require a thorough clinical and laboratory evaluation followed by yearly serology, and a low threshold for reinvestigation [119]. The main value of the IHA remains as a screening test for nonacute melioidosis. In Thailand, an immunochromatographic test (ICT) and IHA were compared on two occasions, using culture as a gold standard and the sensitivity (67–82% and 73% respectively) and specificity (47–80% and 64–68% respectively) of both were low [116,120]. In endemic regions a cut-off titre ≥160 may provide the greatest balance between sensitivity, specificity, and accuracy [118].

Enzyme linked immunosorbent assays (ELISA) detecting IgG and/or IgM have also been used in the serodiagnosis of melioidosis. A number of largely in-house assays together with some commercial assays have been used with varying sensitivities and specificities. One assay developed by Ashdown, had a reported sensitivity of 95% and a specificity of 81%, with no difference noted between acute and convalescent sera [114].

A previously trialled ELISA-based prototype, which utilised an immunochromatographic (ICT) serological method, was assessed by Cuzzubbo et al [121]. This measured both IgG and IgM and utilised a culture filtrate antigen of *B. pseudomallei*. It was shown to have a sensitivity of 100% for IgG and 93% for IgM, with both assays having specificity of 95% [121]. However, when trialled in the field, a commercial version of the assay demonstrated better specificity (97%) but a lower sensitivity (50.6%) for diagnosis of melioidosis [122].

The immunofluorescent antibody (IFA)–IgM has a similar sensitivity and specificity to the ELISA but requires the use of a fluorescent microscope and can be subjective, particularly when used by inexperienced observers [123].

A more recent development has been the use of chimeric antigens. These are fusion proteins from *B. pseudomallei*, for example, rGroEL-FLAG300. When used in an ELISA assay, this showed 95% sensitivity and 95% specificity. Although the sera were from culture-positive patients, no information on time of collection in relation to culture positivity was included in the study [124].

Another promising advance in the serological diagnosis of melioidosis, is the development of the Haemolysin coregulated protein 1 specific ICT (Hcp1-ICT) as the target antigen used for antibody detection, which showed a diagnostic sensitivity and specificity of 88% and 81% respectively, in Thai acute melioidosis patients compared to blood culture [125]. This test has potential as a diagnostic screening test in resource-limited countries in future.

Similarly, in a study evaluating the BpOmpW antigen as a vaccine candidate for melioidosis, antibodies to this antigen have been recognised in the plasma of melioidosis survivors with diabetes [126]. The result aligns with prior challenges in melioidosis diagnostics and therapeutics. While ICT antibody assays like IgG and IgM rapid tests offer moderate diagnostic sensitivity (67–79%) and specificity (80–90%) they are hampered by high background seropositivity in endemic areas. The antibody-detection of the BpOmpW antigen addresses a critical gap by detecting strong cellular and humoral immunity responses [126].

An antibody-detection 4-plex dipstick has been trialled combining four novel antigens namely Hcp1 and BPSL2096, BPSL2697, and BPSS0477. This demonstrated 92% sensitivity and 97–100% specificity for diagnosis of melioidosis [127].

Another antibody-detection ELISA assay that combined 4 antigens used recombinant proteins (TssD-5, Omp3, smBpF4, and Omp85). The recombinant Type VI secretion system protein (TssD-5), achieved 71% sensitivity and 96% specificity. The use of a multiple-antigen ELISA demonstrated better diagnostic sensitivity (88%) while retaining good specificity (96%) than the individual antigens alone [128].

A novel addition to serodiagnosis is the use of antibody-detection assays (ELISAs) based on Hcp1 (Hcp1-ELISA) and O polysaccharide (OPS-ELISA) serology in tandem with real-time PCR based on the type 3 secretion system 1 genes (*TTS1*-PCR), The addition of RT PCR on blood or a clinical sample to the HCP-1 ELISA improved the diagnostic sensitivity and specificity to 98 and 89 respectively [104]. Moreover, among culture-negative patients, 22% were Hcp1-ICT-positive, and subsequent cultures confirmed melioidosis in 31% of these cases, indicating that Hcp1-ICT can identify occult infections missed by culture and PCR alone [103,104]. These studies suggest that integrating molecular and serological approaches is important for early and accurate diagnosis of melioidosis.

The serological diagnosis of melioidosis remains challenging. Clinicians need to be aware of these limitations. It is hoped that the use of novel antigens can improve the sensitivity of serological diagnostics for this condition.

### Laboratory approach in low- and middle-income countries

Understanding the true burden of *B. pseudomallei* in LMIC is challenging due to the absence of well-supported bacterial culture facilities, especially in rural regions where most affected patients likely reside. Diagnostic laboratory services, including microbiology, are often absent or underdeveloped in LMIC and availability of trained personnel can be limited [129]. Varying diagnostic capacity, especially in rural or remote regions, means many cases go undetected, limiting understanding of the disease’s true distribution [130].

As previously described, *B. pseudomallei* requires prolonged incubation, selective media such as Ashdown’s agar for optimal sensitivity for specimens from sites with a normal flora, and specific identification methods. Ashdown’s agar is not commonly used in endemic LMIC. Although cost-effective, it is not commercially available in most countries, requiring reagents, expertise and facilities for in-house production [131]. Many LMIC laboratories have limited access to advanced identification tools such as MALDI-TOF and rely on Analytical Profile Index (API) systems or conventional in-house biochemical tests for bacterial identification, which may be insufficient. While the introduction of EUCAST breakpoints for disc diffusion have improved AST accessibility, reliable access to antibiotic discs and standardised, quality-controlled procedures remains a barrier for many LMIC laboratories [132].

Identification of bacterial isolates begins with specimen culture; a process that many LMIC laboratories should aim to strengthen. Expanding specimen collection is essential—particularly blood cultures for patients with sepsis—and should be complemented by syndrome-specific sampling like sputum or urine when relevant. These foundational improvements lay the groundwork for more accurate and timely diagnosis.

In resource-limited settings, a practical bacterial identification strategy is the use of the three-disc susceptibility test for unidentified, oxidase-positive, Gram-negative bacteria [34]. When reagents are scarce, this approach can be reserved for blood culture isolates and patients presenting with severe pneumonia.

To implement this algorithm effectively in areas with low, uncertain, or suspected melioidosis endemicity, laboratory scientists need training in recognising characteristic Gram-stain features and colony morphology. Equally important is clinician education: communicating clinical suspicion of melioidosis to the laboratory can improve identification rates and enhance lab safety.

Despite the significant burden of *B. pseudomallei* in LMIC, melioidosis diagnosis continues to receive limited global attention, largely due to its minimal impact on high-income, nonendemic countries [133].

### Future directions

The lack of access to high-quality diagnostic instruments and tests is a major contributor to the underdiagnosis and associated morbidity and mortality of melioidosis. The World Health Organization (WHO) has published the ASSURED criteria for the ideal test which can be used at all levels of healthcare in resource-limited settings to guide diagnosis and management of infectious diseases [134]. The hallmarks of this ideal point-of-care test include affordability, accessibility, and accuracy. An expanded REASSURED criteria include connectivity, ease of specimen collection, and provide a reference point for afforbability, Table 2 [135]. Noting that the concentration of *B. pseudomallei* is lowest in blood samples, further efforts should be targeted towards advances in noninvasive samples including sputum and urine [108]. Currently, the lateral flow assay is most likely to fulfil these criteria and improve the diagnosis and management of melioidosis in the majority of endemic regions. The role of rapid molecular diagnosis remains limited. A possible future method may utilise a cartridge-based near-POC instrument, similar to that employed for diagnosis of tuberculosis. Ultimately, this assay would still require specialised equipment, a power supply, robust quality assurance, and will likely remain substantially more expensive [135].

**Table 2:** Performance assessment of laboratory techniques in identifying *B. pseudomallei.*

Criteria	API NE [139]	VITEK 2 [36,139]	LFA [36,85,139]	MS [36,54]	qPCR [36,44]
Real-time connectivity	None	None	Potential	Potential	Potential
Ease of specimen collection	Requires bacterial isolate	Requires bacterial isolate	Blood, urine, sputum, pus	Requires culture-enriched sample	Blood, urine, sputum, pus
Affordable: Instrument cost (USD*)	–	$66k	–	$130–240k	$100–140k^α^
Affordable: Cost per test (USD*)	$10.75	$4.90	$2.00	$0.24	$11.00
Sensitive	37–99%	70–99%	7–100%^	100%^#^	0–100%^
Specific	99%	85–100%	74–100%	90–100%	100%
User-friendly	4 steps	3 steps	3 steps	3 steps	4-5 steps
Rapid & Robust	18-24 h	16-24h	15min	5min	4h
Equipment free	Yes	No	Yes	No	No
Deliverable to end-users	n/a	n/a	Yes	No	No

Adapted from REASSURED criteria & based on the data available at the time of this review. *Australian data (AUD 0.65 = USD 1); ^α^: includes extraction, purification, & thermal cycling instruments; ^: specimen type dependent; ^#^: limited clinical specimen data.

Identifying and confirming the first locally-acquired case of melioidosis in a previously unaffected region marks a vital first step in recognising *B. pseudomallei* as endemic in that area. This discovery should prompt clinicians and laboratory staff to maintain heightened awareness and actively investigate for additional cases.

Importantly, improvement in diagnostic capability must be interwoven with improvement in awareness and education. Melioidiosis is not yet classified as a neglected tropical disease (NTD) by the WHO, but causes a higher burden of diseases and mortality than many recognised NTDs [136–138]. This recognition will assist in raising awareness, funding, research, and ultimately improved diagnostic capacity.

### Summary

Appropriate handling of possible *B. pseudomallei* isolates is essential for laboratory safety, although the risk of laboratory-acquired infection is low. Culture remains the current diagnostic gold standard but is limited by appropriate sample collection and the difficulty of identifying and accessing deep-seated foci of infection, organism isolation with or without selective media, and organism identification. Phenotypic identification of *B. pseudomallei* by biochemical tests, which remains the standard method in many low-resource settings, is plagued by inaccuracy and being superseded in developed nations by mass spectrometry and molecular detection.

The perfect test for the diagnosis of melioidosis is one that is cost-effective, portable, easy to use, and accurately identifies the pathogen directly from a clinical sample. Emerging methods including point-of-care antigen detection and CRISPR-based diagnostic assays have the potential to fulfil these criteria and revolutionise the diagnosis of melioidosis, especially in resource-limited settings.

**Key learning points:**

1. Melioidosis remains under-diagnosed in *B.pseudomallei* endemic regions.
2. Enhanced specimen collection practices are required to improve the sensitivity of culture.
3. The growing adoption of mass spectrometry is expected to improve identification of *B. pseudomallei* isolates.
4. Immunochromatographic lateral flow assays offer a rapid, affordable, and user-friendly diagnostic option for melioidosis, particularly in low-resource settings, while recognising the low sensitivity on direct testing of serum.
5. Emerging technologies like CRISPR-Cas hold promise for improving melioidosis detection through superior sensitivity and specificity.

**Five key papers in the field**

1. Dance DAB, Sihalath S, Rith K, Sengdouangphachanh A, Luangraj M, Vongsouvath M, et al. The cost-effectiveness of the use of selective media for the diagnosis of melioidosis in different settings. PLoS Negl Trop Dis. 2019;13(7):e0007598. Epub 2019/07/16. https://doi.org/10.1371/journal.pntd.0007598. PubMed PMID: 31306412; PubMed Central PMCID: PMCPMC6658006.
2. Trinh TT, Hoang TS, Tran DA, Trinh VT, Göhler A, Nguyen TT, et al. A simple laboratory algorithm for diagnosis of melioidosis in resource-constrained areas: a study from north-central Vietnam. Clin Microbiol Infect. 2018;24(1):84.e1-.e4. https://doi.org/10.1016/j.cmi.2017.07.029.
3. Campbell S, Taylor B, Menouhos D, Hennessy J, Mayo M, Baird R, et al. Performance of MALDI-TOF MS, real-time PCR, antigen detection, and automated biochemical testing for the identification of *Burkholderia pseudomallei*. J Clin Microbiol. 2024;62(10):e0096124. Epub 2024/09/05. https://doi.org/10.1128/jcm.00961-24. PubMed PMID: 39235248; PubMed Central PMCID: PMCPMC11481520.
4. Woods KL, Boutthasavong L, NicFhogartaigh C, Lee SJ, Davong V, AuCoin DP, et al. Evaluation of a Rapid Diagnostic Test for Detection of *Burkholderia pseudomallei* in the Lao People’s Democratic Republic. J Clin Microbiol. 2018;56(7). Epub 2018/05/04. https://doi.org/10.1128/jcm.02002-17. PubMed PMID: 29720430; PubMed Central PMCID: PMCPMC6018328.
5. Karatuna O, Dance DAB, Matuschek E, Åhman J, Turner P, Hopkins J, et al. *Burkholderia pseudomallei* multi-centre study to establish EUCAST MIC and zone diameter distributions and epidemiological cut-off values. Clin Microbiol Infect. 2020. Epub 2020/07/13. https://doi.org/10.1016/j.cmi.2020.07.001. PubMed PMID: 32653660. Pakdeerat S, Boonklang P, Angchagun K, Chomkatekaew C, Apichaidejudom N, Dokket Y, et al. Benchmarking CRISPR-BP34 for point-of-care melioidosis detection in low-income and middle-income countries: a molecular diagnostics study. Lancet Microbe. 2024;5(4):e379-e89. Epub 2024/03/18. https://doi.org/10.1016/s2666-5247(23)00378-6. PubMed PMID: 38493790; PubMed Central PMCID: PMCPMC10990966.

## Supporting information

S1 Table Diagnostic performance summary of referenced studies with sensitivity and specificity data. (DOCX)
