# Supplementary material for: Laboratory diagnosis of melioidosis
Source: PLoS Negl Trop Dis. 2025 Dec 4;19(12):e0013761. doi: 10.1371/journal.pntd.0013761 (PMC12677508; doi:10.1371/journal.pntd.0013761)
Supplement: S1 Table — (DOCX) [file pntd.0013761.s001.docx]

**S1 Table. Diagnostic performance summary of referenced studies with sensitivity and specificity data**

| **Reference** | **Diagnostic assay/s** | **Study design** | **Sample type** | **Sample size** | **Sensitivity %** | **Specificity %** |
| --- | --- | --- | --- | --- | --- | --- |
| 36 Campbell et al. | MS  PCR  LFA  Vitek2 | Retrospective &  Prospective | Bacterial isolates | 43  28 | 100  100  100  70 | 90  100  100  85 |
| 37 Steinmetz et al. | LA | Retrospective | Bacterial isolates | 130 | 100 | 100 |
| 38 Duval et al. | LA | Retrospective | Bacterial isolates | 110 | 99 | 99 |
| 39 Tandhavanant et al. | IFA | Prospective | Various clinical specimen | 88 | 33 – 50* | 99 |
| 40 Smith et al. | LA | Retrospective & prospective | Bacterial isolates | 130 | 100 | 100 |
| 41 Inglis et al. | API 20NE  FAME  LA  PCR | Retrospective | Bacterial isolates | 95 | 37  98  94  100 | 92  83  83  100 |
| 42 Amornchai et al. | API 20NE  LA | Retrospective | Bacterial isolates | 800 | 99  99 | 87  100 |
| 43 Woods et al. | LA  IFA  LFI | Prospective | Various clinical specimen | 23 – 252* | 99^  67-100*  0-99* | 100^  85-100*  91-100* |
| 55 Dulsuk et al. | IFA | Prospective | Positive blood cultures | 545 | 100 | 99 |
| 56 Chantratita et al. | IFA | Prospective | Positive blood cultures | 541 | 97 | 100 |
| 57 Anuntagool et al. | ELISA | Retrospective | Various clinical specimen | 8-35* | 67-88* | 96-100* |
| 67 DeMers et al. | ELISA  LFI | Retrospective | Urine, serum | 73 | 48-59*  6-40* | - |
| 82 Robertson et al. | PCR  LFI | Retrospective | Whole blood | 45 | 20  40 | 100  100 |
| 103 Noparatvarakorn et al. | PCR# | Prospective | Various clinical specimen | 2-65* | 42-100* | 85-100* |
| 104 Noparatvarakorn et al. | PCR  ELISA#  LFI | Prospective | Various clinical specimen | 2-173* | 78  69-71  74 | 100  86-90  90 |
| 114 Ashdown et al. | ELISA  IFA  IHA | Retrospective | Serum | 140 | 79-95^β^  86-95^β^  81-92^β^ | 81-99^β^  81-99^β^  92-100^β^ |
| 116 Cheng et al. | IHA | Prospective | Serum | 275 | 56^β^ | - |
| 117 Appassakij et al. | IHA | Prospective | Serum | 622 | 77^β^ | 92^β^ |
| 119 Cheng et al. | IHA  LFI# | Prospective | Serum | 214 | 73  82-86 | 64  47 |
| 120 Wuthiekanun et al. | IHA  LFI# | Prospective | Serum | 269 | 72  67-79 | 68  80-90 |
| 121 Cuzzubbo et al. | LFI# | Prospective | Serum | 59 | 93-100 | 95 |
| 122 Chuah et al. | IHA  LFI# | Retrospective | Serum | 233 | 76  51-72 | 99  71-97 |
| 123 Ashdown et al. | IHA#  IF#  CF | Prospective | Serum | 120 | 94-100  69-100  100 | 100  100  - |
| 124 Wajanarogana et al. | ELISA | Retrospective | Serum | 220 | 95 | 95 |
| 125 Phokrai et al. | IHA  ELISA  LFI | Retrospective | Serum | 986 | 69  83  88 | 68  95-96  86-100 |
| 127 Wagner et al. | LFI | Retrospective | Serum | 330 | 92 | 97-100 |
| 128 Hara et al. | ELISA# | Retrospective | Serum | 158 | 19-71 | 89-96 |

MS: mass spectrometry; PCR: polymerase chain reaction; LA: latex agglutination; IF: immunofluorescence agglutination; API20 NE: analytical profile index 20 non-Enterobacteriaceae; FAME: fatty acid methyl ester; LFI: lateral flow immunoassay; ELISA: enzyme-linked immunosorbent assay; CF: complement fixation

*varies by sample type; ^ turbid blood culture only; # multiple targets; ^β^ cut-off dependent
